# Implementation of a bundle of care in surgical patients

**DOI:** 10.1186/1753-6561-5-S6-O58

**Published:** 2011-06-29

**Authors:** Y Hendriks, C van Schendel, J Romme, S Alibaks, L van der Laan, R Crolla, J Kluytmans

**Affiliations:** 1 Amphia Hospital Breda, Breda, Netherlands

## Introduction / objectives

Bundles (BUN) have been proposed to improve the process of care. We describe the implementation of a BUN in surgical patients (SP).

## Methods

The Amphia hospital is a large teaching hospital. In the third quarter of 2009 a BUN, defined by the national patient safety initiative, was introduced consisting of 4 components. 1) preoperative hair removal, 2) perioperative antibiotic prophylaxis, 3) normothermia and 4) limit the number of door openings. BUN compliance was measured every quarter by visual observations by dedicated infection control practioners.

## Results

BUN compliance was 10% during the first 4 measurements. A multidisciplinary team analysed the reasons for non-compliance and started a program to improve the adherence. Preoperative hair removal was discouraged and when needed a clipper was used. Hypothermia was prevented by preventing cooling down during the transport of the patient to the operating room and before he entered the operating room. A strong reduction in the number of door-openings was achieved by an intensive campaign that created awareness. All HCWs in the OR were involved to create a culture of safety.

BUN compliance improved gradually over the next three quarters to 60% in the first quarter of 2011.

## Conclusion

At the start of the program the adherence to the components of the BUN was very low. This is not a unique situation as BUN adherence in Dutch hospitals participating in the national program in 2010 was below 10%. After an intensive program that required a change in the behaviour and in the culture of the operating room workers a strong increase was observed. The implementation of a BUN is an effective tool to improve the proces of care and requires a true change in the awareness and behaviour of all HCWs.

## Disclosure of interest

None declared.

